# Immunomodulatory performance of GMP-compliant, clinical-grade mesenchymal stromal cells from four different sources

**DOI:** 10.1016/j.heliyon.2024.e24948

**Published:** 2024-01-19

**Authors:** Mandana Kazem Arki, Kasra Moeinabadi-Bidgoli, Bahareh Niknam, Parvaneh Mohammadi, Moustapha Hassan, Nikoo Hossein-Khannazer, Massoud Vosough

**Affiliations:** a Gastroenterology and Liver Diseases Research Center, Research Institute for Gastroenterology and Liver Diseases, Shahid Beheshti University of Medical Sciences, Tehran, Iran; b Basic and Molecular Epidemiology of Gastroenterology Disorders Research Center, Shahid Beheshti University of Medical Sciences, Tehran, Iran; c Experimental Cancer Medicine, Institution for Laboratory Medicine, Karolinska Institute, 141-83, Stockholm, Sweden; d Department of Tissue Engineering and Applied Cell Sciences, School of Advanced Technologies in Medicine, Shahid Beheshti University of Medical Sciences, Tehran, Iran; e Department of Regenerative Medicine, Cell Science Research Center, Royan Institute for Stem Cell Biology and Technology, ACECR, Tehran, Iran

**Keywords:** Mesenchymal stromal cell, GMP-compliant cells, Clinical-grade cells, Cell therapy, Immunomodulation, Regenerative medicine

## Abstract

Inflammatory and autoimmune diseases are among the most challenging disorders for health care professionals that require systemic immune suppression which associates with various side effects. Mesenchymal stromal cells (MSCs) are capable of regulating immune responses, mainly through paracrine effects and cell-cell contact. Since MSCs are advanced therapy medicinal products (ATMPs), they must follow Good Manufacturing Practice (GMP) regulations to ensure their safety and efficacy. In this study, we evaluated the immunomodulatory effects of GMP-compliant clinical grade MSCs obtained from four different sources (bone marrow, adipose tissue, Wharton’s Jelly, and decidua tissue) on allogeneic peripheral blood mononuclear cells (PBMCs). Our results revealed that WJ-MSCs were the most successful group in inhibiting PBMC proliferation as confirmed by BrdU analysis. Moreover, WJ-MSCs were the strongest group in enhancing the regulatory T cell population of PBMCs. WJ-MSCs also had the highest secretory profile of prostaglandin E2 (PGE-2), anti-inflammatory cytokine, while interleukin-10 (IL-10) secretion was highest in the DS-MSC group. DS-MSCs also had the lowest secretion of IL-12 and IL-17 inflammatory cytokines. Transcriptome analysis revealed that WJ-MSCs had the lowest expression of IL-6, while DS-MSCs were the most potent group in the expression of immunomodulatory factors such as hepatocyte growth factor (HGF) and transforming growth factor-β (TGF- β). Taken together, our results indicated that GMP-compliant Wharton’s Jelly and decidua-derived MSCs showed the best immunomodulatory performance considering paracrine factors.

## 1 Introduction

Mesenchymal stromal cells (MSCs) are a subpopulation of stromal cells which can be used for the treatment of various disorders such as myocardial infarction, fibrosis, and systemic inflammatory diseases [[1], [2], [3]]. Mesenchymal stromal cells possess unique biological properties that make them out as extraordinary therapeutic options for various diseases; these biological properties include: differentiation into diverse mature cells, negligible immunogenicity, induction of tissue regeneration, migration to injury sites, and the potency to modulate immune response [[4], [5], [6]]. As the majority of MSCs are lost within the first 48 h of injection, it has been theorized that the main mechanism of MSC-mediated therapeutic activities is the secretion of immunomodulation mediators, cytokines, and growth factors [7,8]. MSCs regulate immune responses by affecting both innate and adaptive immune cells. MSCs have negative impact on the NK cells proliferation and function. They decrease IFN-γ production and suppress cytotoxicity effects of NK cells mainly through PGE2 and indoleamine 2,3-dioxygenase (IDO). In addition, MSCs abolish granule polarization of NK cells. MSCs also suppress immune responses by hampering the proliferation of CD4^+^ and CD8^+^ T cells and inducing T-reg cells. They inhibit differentiation of naïve CD4^+^ T cells into Th17 and Th1 subsets through interleukin-10 (IL-10) secretion. Moreover, along with IL-10, transforming growth factor beta (TGF-B) and PGE2 produced by MSCs inhibit TH17 differentiation by DC, suppress IL-17 production and improve Treg/TH17 ratio [9]. MSCs have shown promising results in the treatment of various inflammatory and autoimmune diseases such as systemic lupus erythematosus (SLE), graft-versus-host disease (GvHD), anal fistulas in Crohn’s diseases, asthma, and multiple sclerosis (MS) [[10], [11], [12]].

Mesenchymal stromal cells can be derived from almost all tissues including adult and neonatal tissues, but most commonly, MSCs are derived from bone marrow, adipose tissue, dental pulp, placenta, and umbilical cord [13]. It has been elucidated that MSCs from different sources vary in biological properties such as plasticity, proliferation capacity, their paracrine performance, and immunosuppressive function; a phenomenon which could be mainly due to different niches [14].

Mesenchymal stromal cells have become an advanced therapy medicinal product (ATMP) according to European regulation 1394/2007, which means the MSCs that are obligated to meet the Good Manufacturing Practice (GMP) criteria in order to become valid ATMP for clinical use. GMP is a regulation system to ensure that products are consistently produced with equivalent efficacy and minimal safety hazards [15]. In order to obtain GMP- compliant and become clinical grade products, MSCs need to meet criteria in the manufacturing process including donor validation, equipment competency and monitoring, raw materials qualification, technical production processes, and staff organization and hygiene [16]. Although MSCs showed magnificent safety in clinical studies in some cases, their efficacy did not meet the expectations. Immunomodulatory effects of MSCs vary between the different sources in terms of their ability to modulation of immune cells, secretion of anti-inflammatory cytokines, and induction of T-reg cells. Moreover, handling and manufacturing methods including isolation, expansion, storage and final product preparation alter their biological properties. The effects ex vivo manipulation and large-scale manufacturing of MSCs on their immunopotency from distinct sources are poorly studied. Understanding the potency of different clinically grade produced MSCs could help researchers in their future studies. In this study, we investigated and compared the immunomodulatory properties of GMP- compliant MSCs from different sources including bone marrow (BM-MSC), adipose tissue (AD-MSC), Wharton’s Jelly (WJ-MSC), and decidua (DS-MSC).

## 2 Materials and methods

### 2.1 Four different MSCs sources

The current study was approved by the Ethics Committee of the Research Institute for Gastroenterology and Liver Diseases, Shahid Beheshti University of Medical Sciences, Tehran, Iran (Ethical code: IR.SBMU.IRGLD.IEC.1399.038). Different GMP-grade MSCs from various sources including bone marrow (BM-MSC), adipose tissue (AT-MSC), Wharton's Jelly (WJ-MSC), and decidua (DS-MSC), were obtained from Celltech Farmed Company (Tehran, Iran).

Characterization of the MSCs was performed based on the ISCT guidelines. Briefly, three criteria were considered to define MSCs: 1) adherence to plastic 2) specific surface antigen (Ag) expression, and 3) multipotent differentiation potential [17]. The Supplementary Table S1 represents the percentage of the expression of positive and negative selection markers of MSCs from different sources. The left four columns represent positive selection markers and the five right ones represents negative selection markers. In addition, MSCs in defined permissive media directly differentiate into adipocytes, chondrocytes, and osteocytes. Regarding the GMP compliance of MSCs, it should be highlighted that GMP compliance of the mentioned MSCs was approved after external audit by the inspectors of the IrFDA (Iranian Food and Drug Administration) in which all the production process and documentations were checked and validated to verify if they are following PIC/S guide to GMP or not. After validation of the following documents, the GMP certification was issued by the regulatory body. The necessary documentations for GMP compliance were generated in process validation, method validation, cleaning validation, media fill, shipment validation, area qualification, personnel qualification, and qualification and calibration of equipment. All four MSC-banks from each source were developed from a single donor after fulfilling all necessary documents based on the national guidelines of IrFDA. Cells in passage number four used in the experiments.

### 2.2 Peripheral blood mononuclear cell (PBMC) isolation

In order to isolate peripheral blood mononuclear cells (PBMCs), venous blood samples from healthy donors were collected in heparinized tubes. PBMCs were isolated and purified by density gradient centrifuge using the Ficoll-Paque procedure. For this purpose, whole blood was diluted with an equal volume of phosphate-buffered saline (PBS,Sigma-Aldrich,Germany), pH 7.4, gently placed on Ficoll (Biosera, England), and centrifuged at 400 g for 20 min without a break. The PBMC interphase, located between the plasma layer and the Ficoll, was carefully separated by pipetting and then washed with PBS at 200 g for 10 min. A complete culture medium containing RPMI 1640 (Biosera-England) FBS 10% (GIBCO/BRL, Karlsruhe, Germany), 100 Iu/ml penicillin, and 100 μg/ml streptomycin (Biosera, England) was used to resuspense the pelleted cells.

### 2.3 Cell co-culture

To evaluate the immunomodulatory effects of MSCs, including BM-MSC, AD-MSC, WJ-MSC, and DS-MSC, on allogeneic lymphocytes, MSCs and PBMCs were co-cultured with transwell (SPLInsert™ Hanging, SPL, Korea) (0.4 μm) for 72 h. In transwell co-culture, MSCs (4 × 10^4^/well in a 24-well plate) were placed in the lower chamber of the plate 24 h before the addition of purified allogeneic PBMCs (4 × 10^4^ and 5 × 10^5^) with a ratio of 1 : 1 and 1 : 5 in the upper chamber of the plate. A group of PBMCs was stimulated by 1.5% phytohemagglutinin (PHA, Phytohemagglutinin, Gibco, Germany). The Control groups were PBMCs cultured alone, either with or without PHA.

### 2.4 Proliferation assay

In order to investigate the effects of MSCs from different sources on the proliferation of allogeneic PBMCs after 72 h of co-culture, PBMCs in different groups, including control and test, were transferred to a 96 well plate, and the proliferative assessment of PBMCs upon co-culture with allogenic MSCs was performed by 5-Bromo-2′deoxyuridine (BrdU) *(*Roche Diagnostics, Germany) proliferation test according to the manufacturers' recommendation. Briefly, the proliferating cells were labeled by BrdU for 24 h, and after fixing them on the plate, they were stained using a peroxidase-conjugated monoclonal antibody. Finally, after adding the substrate, the absorbance of the samples was measured by a microplate reader spectrophotometer at dual wavelengths of 450/550 nm.

### 2.5 Immunophenotyping of mononuclear cells

72 h after co-culturing PBMCs with MSCs, the percentage of T reg cells, the main cells in modulating and suppressing immune responses, was determined using the flow cytometry technique. The cells were stained with monoclonal antibody conjugated fluorescent dyes according to the instructions of the human flow kit (FOXP3 Alexa Fluor® 488/CD4 APC/CD25 PE). A flow cytometer (BD FACSCalibur™), was used to analyze the stained cells and data analysis was performed using FlowJO.

### 2.6 Cytokine assay

After 72 h of co-culture, conditioned supernatants of different groups were collected to quantify the secreted inflammatory and anti-inflammatory cytokines, including IL-6, IL-10, IL-17 (Invitrogen, Thermo Fisher Scientific,USA), and PGE-2 (R&D Systems, UK),. These measurements were performed using the ELISA kit according to the manufacturer's instructions.

### 2.7 Quantitative real-time PCR

The total RNA was extracted from each group using Trizol reagent (Invitrogen Inc., USA), according to the product's instructions. To quantify RNA, a spectrophotometer (NanoDrop Technologies, USA) was used to measure the absorbance at 260 nm and 280 nm wavelengths. Complementary DNA (cDNA) were synthesized using extracted RNA as template, relative forward and reverse primers, and reverse transcriptase kit according to the manufacturer’s instructions (Yekta Tajhiz Azma, Iran). Real-time quantitative PCR (qRT-PCR) was performed on PCR Rotor-Gene Q real-time PCR Detection System (Qiagen, Hamburg, Germany) using the standard SYBR Green Master Mix Kit (Ampliqon, Odense, Denmark). GAPDH (Glyceraldehyde 3-phosphate dehydrogenase) served as the housekeeping gene. Each sample was performed in triplicate. Primers were as follows, presented in 5′–3′ orientation: HGF-F (AGACCAATGTGCTAATAGATGTA), HGF-R (GCAGTTTCTAATGTAGTCTTTGT), IL-6-F (AACCTGAACCTTCCAAAGATGG), IL-6-R (TCTGGCTTGTTCCTCACTACT), and TGF-β-F (AACCCACAACGAAATCTATGAC), TGF-β-R (TAACTTGAGCCTCAGCAGAC). Statistics were performed on Ct using REST software (Version V2.0.13, Qiagen, Hilden, Germany).

### 2.8 Statical analysis

Statistical analysis and curve fitting were done with GraphPad Prism software version 8 (GraphPad Software, Inc., San Diego, CA, USA). Data from three independent experiments were described as means ± standard deviations. Statistical significance was defined with a one-way analysis of variance after validating the normality and homoscedasticity of the data sets. For all analysis, statistical significance considered as *P* < 0.05.

## 3 Results

### 3.1 MSCs inhibit PBMCs proliferation in both activated and quiescent states

**Fig. 1:**
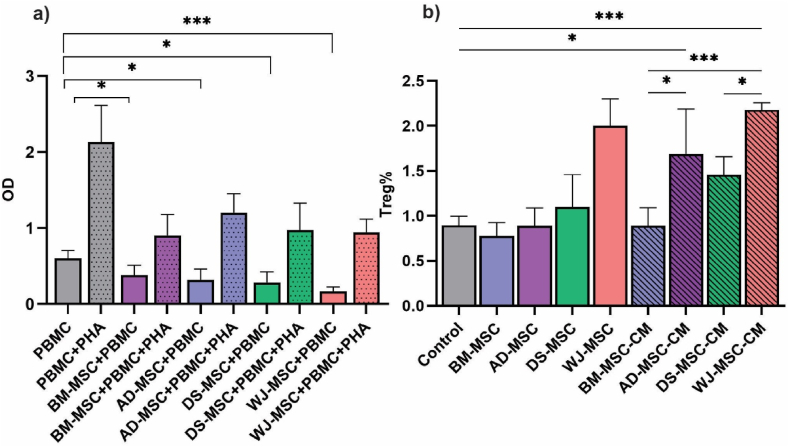
**(a)** BrdU assay of PBMC proliferation upon co-culture with MSCs. The most suppressive effect on PBMC proliferation was instated by WJ-MSCs. Moreover, adding PHA to PBMC culture medium enhanced their proliferation rate**. (b)** T reg population among PBMCs upon co-culture with different MSC groups or their culture medium (CM). T reg population was the highest when PBMCs were co-cultured with WJ-MSC-CM. In addition, MSC-CM induced T reg population more compared to MSCs. This experiment was performed in three biological replicates. All Data resulting from a mean of triplicate experiments were described as means ± SD and statistical significance was considered as *P* ≤ 0.05. (*P < 0.05, **P < 0.01, and ***P < 0.001).

BrdU is an analog of thymine nucleotide used to assess the proliferation of cells via DNA labeling during the S-phase of the cell cycle. Hampering the proliferation of immune cells has a vigorous impact on inhibiting inflammatory responses and prohibiting immune-mediated tissue damages. As shown in Fig. 1, WJ-MSC + PBMC group followed by DS-MSC + PBMC, AD-MSC + PBMC, and BM-MSC + PBMC groups, had the lowest proliferation rate, respectively, compared to the control (PBMC alone) in a statistically significant manner (*p*-value 0.0004, 0.121, 0.0192, 0.040). It was shown that despite adding PHA to PBMCs, MSCs suppressed their proliferation. Among PHA-activated PBMCs, BM-MSC + PBMC + PHA group had the lowest proliferation rate, followed by WJ-MSC + PBMC + PHA, DS-MSC + PBMC + PHA, and AD-MSC + PBMC + PHA groups respectively. All differences were statistically significant (*p*-value <0.05). Fig. 1a represents BrdU results in different group (Fig. 1a).

### 3.2 MSCs and their culture medium increased T reg population of PBMCs

To measure T reg populations in response to co-culture with MSCs, PBMCs were exposed to different MSC groups and their culture medium, then the T reg populations were measured by flow cytometry. As shown in Fig. 1, it was observed that PBMCs cultured with WJ-MSC-CM induced the highest percentage of T reg population (2.1%, statically significant (*p*-value 0.001)) followed by WJ-MSC (1.9%), AD-MSC-CM (1.7%, statically significant *p*-value 0.026), DS-MSC-CM (1.5%, *p*-value 0.045), DS-MSC (1.2%), AD-MSC and BM-MSC-CM (0.8%) groups, respectively. Taken together, it was elucidated that the culture medium of MSCs had a more potent effect in inducing T reg population than their co-culture, and WJ-MSCs were the most ideal group in induction of T reg population in PBMCs. Fig. 1b represents T reg population results after treatment in different group.

### 3.3 MSCs enhanced IL-10 and PGE-2 secretion from PBMCs while suppressing IL-6, IL-12, and IL-17

**Fig. 2:**
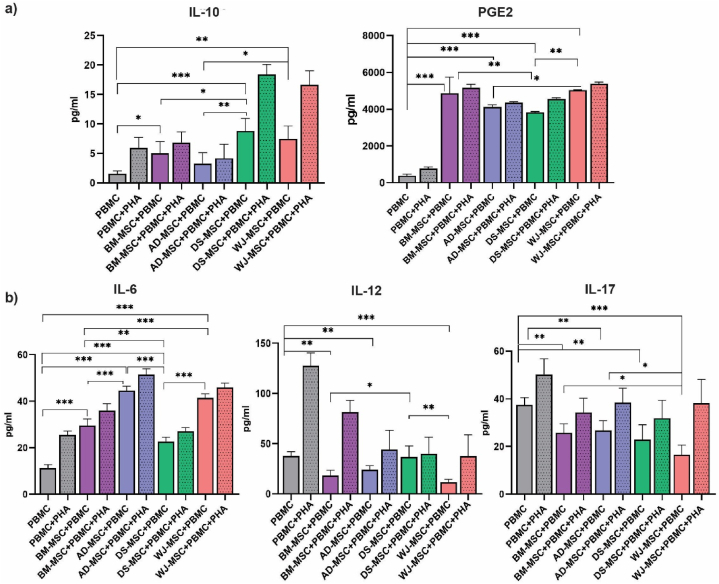
IL-10, IL-12, IL-6, PGE2, and IL-17 secretory profile upon PBMC co-culture with MSCs. WJ-MSC and DS-MSC, compared to other groups increased more IL-10 levels. WJ-MSC and BM-MSC secreted the highest levels of PGE2 (a). On the other hand, WJ-MSC and BM-MSC decreased the production of IL-17 and IL-12 inflammatory cytokines by PBMC (b). IL-6 increased significantly in AD-MSC and WJ-MSC co-culture groups compared to other groups (b). This experiment was performed in three biological replicates. All Data resulting from a mean of triplicate experiments were described as means ± SD and statistical significance was considered as *P* ≤ 0.05. (*P < 0.05, **P < 0.01, and ***P < 0.001).

In order to assay the secretion of cytokines from PBMCs upon co-culture with MSCs, ELISA assay was utilized. The values of IL-10 and PGE2 as anti-inflammatory cytokine and IL-6, IL-12, and IL-17 inflammatory cytokines were measured and are shown in Fig. 2a and b. In our experiment, DS-MSC + PBMC + PHA group and WJ-MSC + PBMC + PHA group displayed a significant increase (*p*-value <0.05) in IL-10 secretion compared to the control group (PBMC + PHA). Among PHA negative groups, DS-MSC + PBMC, WJ-MSC + PBMC, and BM-MSC + PBMC groups showed significant (*p*-value: 0.0005, 0.0019, 0.013) secretion of IL-10 [18]. It was demonstrated that while AD-MSC + PBMC +/− groups had the highest IL-6 secretion, DS-MSC + PBMC +/−, BM-MSC + PBMC +/− PHA, and WJ-MSC + PBMC +/− PHA groups had the lowest secretion of IL-6, respectively. We observed that WJ-MSC + PBMC group had the lowest IL-12 secretion profile (statically significant (*p*-value 0.0001)), followed by BM-MSC + PBMC (statically significant (*p*-value 0.001)) and AD-MSC + PBMC (statically significant (*p*-value 0.002)), respectively. Our study showed that WJ-MSC + PBMC group demonstrated a significant (*p*-value 0.0002) reduction in IL-17 production, followed by DS-MSC + PBMC (statically significant (*p*-value 0.0057)), BM-MSC + PBMC, and AD-MSC + PBMC groups. Our study elucidated that all the MSC groups are able to induce PGE-2 production in PBMCs, regardless of PHA addition. WJ-MSC + PBMC + PHA and BM-MSC + PBMC + PHA groups displayed the highest PGE-2 secretion, followed by WJ-MSC + PBMC, BM-MSC + PBMC, DS-MSC + PBMC + PHA, AD-MSC + PBMC + PHA, AD-MSC + PBMC, and AD-MSC + PBMC groups. All results were statically significant (*p*-value <0.05). It should be mentioned that values of the cytokine release profile (PGE2, IL10, IL6, and IL17) in the co-culture groups were presented after subtracting the cytokine values generated by MSCs alone from the total cytokine values. These values indicate the values of cytokines generated by PBMCs. The amounts of cytokines produced by MSCs alone were not presented in the graphs due to the data and space limitations.

Taken together, it has been elucidated that MSCs, especially WJ-MSCs and DS-MSCs are able to regulate the secretion of cytokines such as IL-6, IL-10, IL-12, IL-17, and PGE-2 from PBMCs to modulate immune system activity.

### 3.4 DS-MSCs induced genes involved in immunomodulation more than other groups

**Fig. 3:**
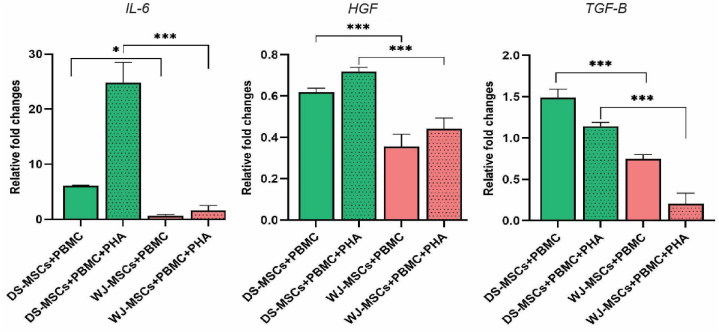
Relative *IL-6*, *HGF*, and *TGF-β* expressions of PBMCs upon co-culture with DS-MSC and WJ-MSC groups. Addition of PHA augmented IL-6 expression in both DS-MSC and WJ-MSC. IL-6 expression was the lowest in WJ-MSC group compared to others. Addition of PHA decreased HGF expression in both DS-MSC and WJ-MSC. HGF expression was significantly higher in DS-MSC group compared to other groups. Addition of PHA enhanced TGF-β expression in both DS-MSC and WJ-MSC. TGF-β expression was significantly higher in DS-MSC-PHA group compared to others. This experiment was performed in three biological replicates. All Data resulting from a mean of triplicate experiments were described as means ± SD and statistical significance was considered as *P* ≤ 0.05. (*P < 0.05, **P < 0.01, and ***P < 0.001).

Quantitative real time PCR (qRT-PCR) was conducted to evaluate the expression of genes involved in immunomodulatory activities including IL-6, HGF, and TGF-β. Relative gene expression assay was performed by comparing MSCs co-cultured with PBMCs (±PHA) with the control groups (DS-MSC and WJ-MSC). It was demonstrated that WJ-MSC + PBMC group had the lowest expression profile of IL-6, followed by WJ-MSC + PBMC, DS-MSC + PBMC, and DS-MSC + PBMC + PHA groups respectively (Fig. 3). DS-MSC + PBMC group had the most profound upregulation of HGF expression, followed by DS-MSCs + PBMC + PHA, WJ-MSC + PBMC, and WJ-MSC + PBMC + PHA groups. It was also observed that the DS-MSC + PBMC + PHA group demonstrated the highest TGF-β expression, followed by DS-MSC + PBMC, WJ-MSC + PBMC + PHA, and WJ-MSC + PBMC groups, respectively.

Taken together, our experiment show that while GMP-grade WJ-MSCs had the lowest IL-6 expression, GMP-grade DS-MSCs had the highest expression level of HGF and TGF-β.

## 4 Discussion

After years of investigation, it has now become evident that MSCs have the capacity to be applied in various clinical scenarios such as ischemic disorders, malignancies, and autoimmune diseases [19,20]. Increasing the use of MSCs in humans as advanced therapy medicinal products (ATMPs), as described by Europeans Medicines Agency, necessitates the accordance of their production and preparation processes with GMP regulations. Donor heterogeneity is one of the main causes of the discrepancies in MSC’s therapeutic capacity as various MSCs’ biological properties depend on donor’s condition. For instance, MSC obtained from different donors vary in their secretory profile [21]. It has been shown that donor’s sex affects the immunomodulatory properties of MSCs as female-derived MSCs showed higher suppression of PBMCs’ proliferation compared with male-derived MSCs [22]. Donor’s age is also an important factor. It has been demonstrated that MSC obtained from older donors have impaired proliferative and osteogenic potential compared with MSCs obtained from younger donors [23]. According to GMP regulations, donors should be healthy and have no risk of abnormalities; moreover, MSC isolation techniques are described for various MSC sources to minimize heterogeneity [16,24]. MSC culture protocols are also required to follow GMP regulations to obtain sufficient cell population and to avoid heterogeneity or inadequate therapeutic efficacy. GMP regulations also describe standard culture and isolation equipment, techniques, protocols as well as proper staff training and qualification to avoid microbial contamination and minimize the risk of genomic instability [25]. Preparing MSCs according to GMP regulations will reduce their heterogeneity and enhance their paracrine activity and therapeutic effects [26]. As mentioned earlier, MSCs could be derived from different sources with different biological properties. MSCs from different sources are diverse in the quality and quantity of paracrine factors including cytokines, growth factors, and EVs’ content [27]. Moreover, secretion of anti-inflammatory cytokines is dissimilar among MSCs from different sources [28]. MSC’s source is a critical determinant for their differentiation capacity. It has been shown that differentiation of BM-MSC and AD-MSC toward adipose tissue is higher compared with umbilical cord-derived MSCs [29]. It has been revealed that MSC derived olfactory mucosa have greater proliferation rate and differentiation capacity into dopaminergic neurons compared to WJ-MSC [30]. While some studies have compared the immunomodulatory properties of GMP-grade MSCs from different sources, our experiment is the first study to include GMP- compliant DS-MSCs and evaluate the impact of MSC co-culture on the secretory and gene expression profile of PBMCs [[31], [32], [33]]. In this study, we used GMP- compliant MSC’s from four different sources: bone marrow, adipose tissue, decidua, and Wharton’s Jelly, to approximate the results to clinical practice.

Immunomodulatory and anti-inflammatory properties of MSCs have been approved in multiple *in vitro* and *in vivo* studies and have been brought up as one of the main therapeutic features of MSCs [34]. The main goal of this study is to determine the best source for MSC isolation to obtain the maximum efficacy with respect to immunomodulatory activity.

Immune cells expand and undergo extensive proliferation in response to activation and pro-inflammatory microenvironment [35]. In multiple studies, inhibiting PBMCs’ proliferation is suggested as one the main means of inflammation suppression [36]. BrdU results revealed that all four GMP- compliant MSC types are capable of suppressing PBMCs expansion, with WJ-MSCs being the most potent type.

Regulatory T cells modulate immune system activity and diminish inflammatory responses [37]. T reg immunomodulatory features have been proposed as being beneficial for the treatment of various autoimmune disorders such as psoriasis [38,39]. It has been shown that T regs secrete various paracrine cytokines including IL-10, TGF-® and IL-35 to modulate the immune system’s activity in the area of inflammation [40]. In this experiment, we have observed that the culture medium of WJ-MSC, DS-MSC, and AD-MSC as well as using co-culture system could significantly enhance T reg cells in the PBMCs population, likely through the enhanced secretion of IL-10 [41].

It is now evident that MSCs suppress inflammation and immune cells activity through their paracrine function and secretion of growth factors and cytokines [42]. Moreover, paracrine activity and secretion of cytokines are the main tools of immune cells to regulate inflammation [43]. Immunosuppressive activity of MSCs mostly relies on paracrine effects and their secretome. The majority of the therapeutic effects of MSCs occur through secretion of cytokines, growth factors, soluble mediators, and extracellular vesicles [44]. Conditioned medium of MSCs had a more potent effect in inducing T reg population than their co-culture [45]. IL-10 is a key factor in inhibiting inflammatory response and plays a pivotal role in regulating immune responses and preventing autoimmunity [46]. Our results demonstrated that DS-MSCs are the most potent inducers of PBMCs IL-10 secretion. PGE-2 is an essential regulator of immune system function, since the dysregulated PGE-2 production and secretion are attributed to certain autoimmune disorders [47]. In our experiment, it was revealed that WJ-MSCs and BM-MSCs are a significant cause of PGE-2 secretion by PBMCs. It is now evident that PGE-2 induces the secretion of IL-10. It is possible that the enhanced secretion of IL-10 in our survey could be attributable to the increase in PGE-2 secretion. The discrepancy among the groups with the highest IL-10 and PGE-2 secretion (DS-MSC group for IL-10 and WJ-MSC and BM-MSC groups for PGE-2) could be explained by different translational or post-translational processes [48]. IL-6 is a key player in immune homeostasis and inflammatory response induction and maintenance of chronic inflammation [49]. We have noticed that the DS-MSC group had higher IL-6 expression compared with the WJ-MSC group, IL-6 secretion was lower in the DS-MSC group in the ELISA assay, possibly due to different translational or post-translational modifications or transport processes. IL-12 and IL-17 pro-inflammatory cytokines are crucial in immune responses in autoimmune diseases, thus, hampering their secretion is beneficent in the treatment of autoimmunity [50,51]. Our result data elucidated that WJ-MSCs are the most potent group in suppressing IL-12 and IL-17 secretion from PBMCs, possibly in a PGE-2 dependent manner.

To further validate the results, we conducted a gene expression assay for some paracrine factors from MSCs. It was shown that WJ-MSCs had the lowest expression of IL-6 upon co-culture with PBMCs. TGF-β is a pivotal factor in instating immune tolerance and autoimmunity prevention [52]. HGF is a growth factor capable of preventing autoimmunity progression by regulating pathophysiological processes such as immune cell migration, antigen presentation, and cytokine production [53]. Our data revealed that DS-MSCs possess the highest expression profile of TGF-β and HGF upon co-culture with PBMCs.

Finally, recent studies showed that preconditioning of MSCs induce their survival and proliferation rates as well as immunomodulatory effects. In vitro preconditioning strategies including hypoxic/anoxic culture condition, adding growth factors, cytokines, chemical agents, and lipopolysaccharide (LPS) to the culture medium induce different signaling pathways and enhance paracrine activity of MSCs. So, it is proposed that application of MSCs preconditioning may improve their therapeutic effects [54].

## 5 Conclusion

In conclusion, we have investigated the immunomodulatory properties of MSCs from different sources upon co-culture with PBMCs. It was elucidated that among the investigated GMP-compliant clinical grade MSCs, DS-MSCs and WJ-MSCs are the most potent cells in regulating inflammatory responses and inducing immunomodulation in PBMCs with respect to hampering PBMC proliferation, inducing T reg population, modulating secretion of cytokines, and expression of the genes involved in the regulation of inflammation. Although these results require further confirmation in animal studies and clinical trials, our study could be a step-forward in management of practical application of MSCs for the treatment of immune-mediated disorders.

## Ethics statement

This study was approved by the Ethics Committee of the Research Institute for Gastroenterology and Liver Diseases, Shahid Beheshti University of Medical Sciences, Tehran, Iran (Ethical code: IR.SBMU.IRGLD.IEC.1399.038). Different GMP-grade MSCs from various sources including bone marrow (BM-MSC), adipose tissue (AT-MSC), Wharton's Jelly (WJ-MSC), and decidua tissue (DS-MSC), were obtained from CellTech Pharmed Company (Tehran, Iran). This company works under regulation of IrFDA and they have informed consent and ethical approval for any bio-bank.

## Funding

This project supported by grant form “10.13039/501100015693The Research Institute for Gastroenterology & Liver Diseases, SBUMS” Iran. grant No. 24697.

## CRediT authorship contribution statement

**Mandana Kazem Arki:** Writing – original draft. **Kasra Moeinabadi-Bidgoli:** Resources, Investigation. **Bahareh Niknam:** Methodology, Investigation, Conceptualization. **Parvaneh Mohammadi:** Validation, Methodology, Data curation. **Moustapha Hassan:** Writing – review & editing, Visualization. **Nikoo Hossein-Khannazer:** Supervision, Project administration. **Massoud Vosough:** Writing – review & editing, Supervision.

## Declaration of competing interest

MV is the regulatory affairs manager at CellTech Pharmed. He doesn’t have any share in this company. Authors declare that they have no conflict on interests.
