# Supplementary material for: Immunomodulatory performance of GMP-compliant, clinical-grade mesenchymal stromal cells from four different sources
Source: Heliyon. 2024 Jan 19;10(2):e24948. doi: 10.1016/j.heliyon.2024.e24948 (PMC10835001; doi:10.1016/j.heliyon.2024.e24948)
Supplement: Multimedia component 1 [file mmc1.docx]

**Table S1. GMP-COMPLIANT MSCs CHARACTERIZATION BASED ON SURFACE MARKERS (%) by Attune^TM^ NxT Cytometer (n=3 replicates)**

| **MSC Source** | **CD73** | **CD90** | **CD105** | **CD29** |  | **CD45** | **CD34** | **CD11b** | **CD31** | **HLA-DR** |
| --- | --- | --- | --- | --- | --- | --- | --- | --- | --- | --- |
| **Bone Marrow** | 99.6 ± 0.3 | 99.8 ± 0.2 | 99.6 ± 0.4 | - |  | 0.06 ± 0.01 | 0.04 ± 0.03 | - | - | 0.06 ± 0.05 |
| **Adipose Tissue** | 99.9 ± 0.1 | 100 | 99.9 ± 0.1 | 100 |  | 0.43 ± 0.12 | 0.59 ± 0.12 | 0.62 ± 0.08 | 0.17 ± 0.07 | 0.02 ± 0.01 |
| **Wharton’s Jelly** | 100 | 99.9 ± 0.1 | 99.4 ± 0.3 | 100 |  | 0.24 ± 0.14 | 0.86 ± 0.08 | 0.20 ± 0.04 | - | 0.1 ± 0.03 |
| **Decidua Tissue** | 96.6 ± 0.04 | 100 | 99.9 ± 0.10 | 99.2 |  | 0.18 ± 0.15 | 0.30 ± 0.09 | 0.56 ± 0.26 | 0.00 | 0.24 ± 0.02 |
